# A Case of Sciatica During Labor Due to an Occiput Posterior Fetus

**DOI:** 10.7759/cureus.2082

**Published:** 2018-01-17

**Authors:** Cassandra Wasson, Telianne Chon

**Affiliations:** 1 Anesthesia, Riverside University Health System Medical Center, Moreno Valley, California, United States

**Keywords:** occiput posterior, epidural breakthrough pain, fetal malposition, sciatica

## Abstract

A 28-year-old term G3P0020 received an epidural with complete pain relief. Approximately 19 hours after the epidural placement, the pain increased. Sensory levels were rechecked and were bilateral and adequate at T8. Further discussion revealed that the pain was unrelated to her contractions; it was in her buttocks and radiating down the leg. The possibility of the fetus being positioned occiput posterior (OP) was discussed. The patient was placed into knee-chest position with instantaneous relief of her pain. This is the only known case report of epidural breakthrough pain due to an OP fetal malposition with successful intra-partum pain management solely by position change.

## Introduction

According to the National Vital Statistics system from the Centers for Disease Control (CDC), 61% of patients in the United States elect to have labor anesthesia via epidural catheters [1]. There have been multiple cases of inadequate pain relief occurring during labor epidurals. This can be very frustrating for the patient, the obstetric nurses, and the anesthesiologists. The ability to determine the cause of the breakthrough pain and evaluate whether the epidural should be adjusted or replaced is imperative to relieve the patient’s labor pain.

## Case presentation

A 28-year-old G3P0020 was admitted to the labor and delivery floor for labor management. An epidural was placed with complete pain relief. Nineteen hours post-epidural placement, the anesthesia team was called to the patient’s bedside because the patient was experiencing increased pain. At that time, the patient had dilated to only 4 cm despite augmentation with oxytocin. The epidural levels were checked and were bilateral and adequate at T8. The patient was informed the epidural was still in the correct position and functional. She agreed, stating she was not feeling contraction pain. Further discussion with the patient revealed her pain was solely in her buttocks and was radiating down the back of her legs, similar to the descriptions of sciatica. The anesthesia team considered the possibility of the baby being occiput posterior (OP) and subsequently applying pressure on the sacral plexus. We discussed this with the nurse, obstetric team, and the patient. The patient was placed in knee-chest position, which caused instantaneous pain relief from a 10/10 to 5/10. Over the next hour, it gradually decreased to 0/10 pain. After five additional hours of labor, the patient delivered a healthy baby girl.

## Discussion

A T10-L1 block is necessary for uterine contractions and cervical dilation, which is important to block the pain during the first stage of labor (cervical dilation and effacement). During the second stage of labor (the actual delivery of the fetus), an S2-S4 block is necessary for vaginal and perineal distention pain [2]. There are many predictors of epidural breakthrough pain, including nulliparity, heavy fetal weight, and epidural catheter placement at an early cervical dilation [3]. These predictors are also the same predictors of increased maternal pain [3]. Our patient had two of these, nulliparity and epidural placement at an early cervical dilation. If breakthrough pain occurs with an epidural, evaluation of the epidural must occur in the cephalad and caudad directions. If no block is present, the epidural catheter needs to be replaced. If the block is inadequate, the epidural needs to be bolused and the rate should be increased. If an adequate block is present, as was in our patient, other causes must be considered [2].

Our patient likely had an OP fetus. This abnormal presentation occurs in approximately 25% of cephalic deliveries [4]. An OP presentation increases maternal discomfort due to the pressure on the lumbosacral plexus; it may cause prolonged labor [4-6]. An OP presentation decreases the rate of a spontaneous vaginal delivery to 26% of nulliparas and 57% of multiparas [5]. The knee-chest position (a prone position with the individual resting on the knees and upper part of the chest) helps relieve the pain [4,6] and may help facilitate the rotation of the fetus to occiput anterior position [4], increasing the rate of a spontaneous vaginal delivery, which did occur with our nulliparous patient. As this is a simple intervention with little risk, it should be tried routinely in patients with new onset of pain amidst an adequate epidural block.

## Conclusions

In conclusion, it is important to evaluate an epidural prior to simply bolusing it or replacing it. Doing either of these two options would not have treated the patient’s pain. Rather, it may have subjected her to an unnecessary procedure and its associated risks without incurring any benefits from it. A discussion with the patient, to further clarify her pain, led to the treatment with position changes, which corrected the fetal malpresentation, relieved her pain, and allowed labor to progress to a successful delivery.

## References

[1] Osterman MJK, Martin JA (2011). Epidural and spinal anesthesia use during labor: 27-state reporting area, 2008. Natl Vital Stat Rep.

[2] Chestnut DH, Wong CA, Tsen LC, Ngan Kee WD, Beilin Y, Mhyre JM (2009). Chestnut's Obstetric Anesthesia: Principals and Practice.

[3] Hess PE, Pratt SD, Lucas TP, Miller CG, Corbett T, Oriol N, Sarna MC (2001). Predictors of breakthrough pain during labor epidural analgesia. Anesth Analg.

[4] Guittier MJ, Othenin-Girard V, de Gasquet B, Irion O, Boulvain M (2016). Maternal positioning to correct occiput posterior fetal position during the first stage of labour: a randomized controlled trial. BJOG.

[5] Ponkey SE, Cohen AP, Heffner LJ, Lieberman E (2003). Persistent fetal occiput posterior position: obstetric outcomes. Obstet Gynecol.

[6] Hunter S, Hofmeyr GJ, Kulier R (2007). Hands and knees posture in late pregnancy or labour for fetal malposition (lateral or posterior). Cochrane Database Syst Rev.

